# Smoking status combined with tumor mutational burden as a prognosis predictor for combination immune checkpoint inhibitor therapy in non‐small cell lung cancer

**DOI:** 10.1002/cam4.4197

**Published:** 2021-09-01

**Authors:** Li‐Yue Sun, Wen‐Jian Cen, Wen‐Ting Tang, Ya‐Kang Long, Xin‐Hua Yang, Xiao‐Meng Ji, Jiao‐Jiao Yang, Ren‐Jing Zhang, Fang Wang, Jian‐Yong Shao, Zi‐Ming Du

**Affiliations:** ^1^ State Key Laboratory of Oncology in South China Guangzhou China; ^2^ Collaborative Innovation Center for Cancer Medicine Guangzhou China; ^3^ Department of Molecular Diagnostics Sun Yat‐sen University Cancer Center Guangzhou China

**Keywords:** combination therapy, immune checkpoint inhibitor, non‐small cell lung cancer, smoking, tumor mutational burden

## Abstract

**Background:**

This study aimed to explore the prognostic value of tumor mutational burden (TMB) combined with smoking status in advanced non‐small cell lung cancer (NSCLC) patients who received immune checkpoint inhibitor therapy (anti PD‐1/PD‐L1 therapy) combined with chemotherapy or anti‐angiogenesis therapy.

**Methods:**

We conducted a retrospective analysis of NSCLC patients who underwent next‐generation sequencing test (either 295‐gene panel NGS or 1021‐gene panel NGS) from September 2017 to November 2020. The relationship between TMB and smoking status was investigated. Kaplan–Meier survival analysis was used to compare progression‐free survival (PFS) of the NSCLC patients who received combination immunotherapy grouped by TMB value and smoking status.

**Results:**

We enrolled 323 cases and 388 cases of NSCLC patients in the 295‐gene panel cohort and 1021‐gene panel cohort, respectively. Positive correlation between TMB and smoking status was found in lung adenocarcinoma, but not in lung squamous cell carcinoma. Participants with both high TMB and smoking status who received immune checkpoint therapy combined with chemotherapy or anti‐angiogenesis therapy had longer PFS than other participants (*p* < 0.05).

**Conclusions:**

The combination of TMB with smoking status might be a potential predictor for the efficacy of combination immunotherapy in advanced NSCLC.

## Graphical abstract

The tumor mutational burden (TMB) value correlated with smoking status in lung adenocarcinoma patients, but not in lung squamous cell carcinoma patients in both NGS panels. The TMB value combined with smoking status might be used as a potential prognostic indicator for advanced non‐small cell lung cancer patients receiving immune checkpoint inhibitor combination therapy.

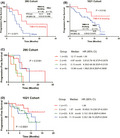

## 1 INTRODUCTION

Immune checkpoint inhibitors (ICIs) for programmed cell death 1 (PD‐1)/programmed cell death‐ligand 1 (PD‐L1) blockade have become one of the effective treatments for advanced non‐small cell lung cancer (NSCLC).^1^ Tumor mutational burden (TMB) has been shown to predict the efficacy of ICI in the clinical studies of CheckMate 026^2^ and CheckMate 227^3^ in NSCLC, but it was controversial in KEYNOTE‐021^4^/158^5^/189^6^/407^7^ studies. Therefore, the predictive value of TMB for ICI treatment in NSCLC still needs further investigation. In addition, due to the lack of standardized detection procedures of TMB, the definition of TMB high from different next‐generation sequencing (NGS) platforms needs further clinical investigation and comparison as well.

Currently the combination of ICI with chemotherapy or anti‐angiogenesis therapy has become a hotspot in the clinical treatment of advanced NSCLC. Some patients have benefited from such combination therapy, including the patients with low PD‐L1 expression^6^, ^7^, ^8^; however, the clinical efficacy prediction markers are lacking for such combination therapy.^9^ Further study is needed to determine if TMB could predict the clinical efficacy in NSCLC patients receiving ICI combined with chemotherapy or anti‐angiogenesis therapy.

Smoking is well known as the primary cause of NSCLC.^10^ However, the correlation between smoking history and TMB is still contentious, and its role in efficacy prediction in ICI treatment of NSCLC needs additional study as well.^11^, ^12^ One study showed that NSCLC patients who smoked could benefit more from second‐line immunotherapy than non‐smoking patients after resistance to first‐line targeted therapy.^13^ Therefore, the relationship between smoking status and TMB, and the predictive value of combining smoking status with TMB for combination immunotherapy in NSCLC still needs further investigation.

In the present study, two advanced NSCLC cohorts from our hospital who received either 295‐gene panel NGS test or 1021‐gene panel NGS test were utilized to analyze the correlation between the smoking status and TMB and their roles in efficacy prediction in NSCLC patients who received ICI combined chemotherapy or anti‐angiogenesis therapy.

## 2 MATERIALS AND METHODS

### 2.1 Patient enrollment

This study retrospectively analyzed NSCLC patients who received either 295‐gene panel NGS test (295 cohort) or 1021‐gene panel NGS test (1021 cohort) at Sun Yat‐sen University Cancer Center from September 2017 to November 2020. The inclusion criteria included pathologically confirmed NSCLC, complete clinical information, and successful NGS detection. The Clinical Research Ethics Committee approved this study with ID: B2020‐344‐01. All procedures of this study were strictly following the Declaration of Helsinki (as revised in 2013).

### 2.2 TMB detection

Tumor mutational burden value was calculated from the high‐throughput sequencing data from both 295‐gene panel and 1021‐gene panel.^14^, ^15^ The gene list for each panel is listed in Table S2 and S3. The NGS library preparation and sequencing protocol were performed as follows. In brief, the genomic DNA was fragmented by Covaris M220 focused ultrasonicator (Covaris, Inc.), converted to an NGS library by end repair, A‐tailing, and adapter ligation. Then, DNA library was purified and quantified using Qubit 2.0 fluorimeter with the dsDNA high‐sensitivity assay kit (Life Technologies). The samples tested with 295‐gene panel were indexed and sequenced on Nextseq500 (Illumina, Inc.) and 1021‐gene panel indexed samples were sequenced on Gene+Seq‐2000 (Geneplus‐Beijing Institute) with paired‐end reads. In both 295‐gene and 1021‐gene panels, TMB was calculated by the number of somatic missense mutations, nonsense mutations, and coding indels and displayed as the number of mutations per Mb of captured genome. Fusions, copy number variations, and non‐coding mutations were not counted. According to x‐tile software,^16^ the optimal cutoff value used to define high TMB is 6.1 mutations/Mb in 295‐gene panel and 15.4 mutations/Mb in 1021‐gene panel, respectively.

### 2.3 Follow‐up

The clinical information of all patients was retrospectively obtained from their medical records. Treatment efficacy was evaluated according to the response evaluation criteria in solid tumors (RECIST) 1.1 criteria. The endpoint of follow‐up was the last visit or disease progression after participants who received combination immunotherapy, and the end point of this study was progression‐free survival (PFS).

### 2.4 Statistical analysis

Statistical analysis was performed using the statistical software SPSS 20.0 (IBM Corp.). Measurement data were expressed as means ± SD, and comparisons between groups were performed by *t*‐test. A *p* < 0.05 was considered to be statistically significant. One‐way ANOVA was used between the three groups of data, and *p* < 0.05 was considered to be statistically different. Kaplan–Meier analysis was used to estimate PFS after receiving combination therapy. GraphPad Prism 7.0 (GraphPad Software) was used for graphing.

## 3 RESULTS

### 3.1 Study population

This study enrolled 323 cases of NSCLC patients in the 295 cohort and 388 cases in the 1021 cohort. The characteristics of participants in the two cohorts are displayed in Table 1. Among them, there were 32 and 58 patients from 295 cohort and 1021 cohort, respectively, who received ICI combined with chemotherapy or anti‐angiogenesis therapy (detailed information of these patients is shown in the Table S1).

**TABLE 1:** Clinical characteristics of NSCLC patients

Characteristic	295 cohort	1021 cohort	*p* value
Number	323	388	
Age (year)	59.60 ± 11.77	58.96 ± 10.84	0.428
Gender, *n* (%)			0.195
Male	206 (63.8)	229 (59.0)	
Female	117 (36.2)	159 (41.0)	
Histology, *n* (%)			0.759
Adenocarcinoma	272 (78.8)	328 (84.5)	
Squamous cell	45 (13.9)	50 (12.9)	
Adenosquamous	6 (1.9)	10 (2.6)	
TNM stage, *n* (%)			**0.000**
I	16 (5.0)	61 (15.7)	
II	10 (3.1)	10 (2.6)	
III	38 (11.8)	69 (17.8)	
IV	247 (76.5)	245 (63.1)	
Unknown	12 (3.7)	3 (0.8)	
Smoking status			**0.000**
Current	100 (31.0)	122 (31.4)	
Never	161 (49.8)	240 (61.9)	
Former	23 (7.1)	26 (6.7)	
Unknown	39 (12.1)	0 (0.0)	
Smoking index (pack × year)	18.92 ± 27.33	15.48 ± 25.93	**0.041**
Prior lines of therapy, *n* (%)			0.127
0	249 (77.1)	278 (71.6)	
1	39 (12.1)	48 (12.4)	
≥2	35 (10.8)	62 (16.0)	
TMB (Muts/Mb)	8.79 ± 7.73	7.17 ± 6.86	**0.042**

Significance of bold means *p* value < 0.05.

Abbreviations: TMB, tumor mutational burden; TNM, tumor, node, metastasis; NSCLC, non‐small cell lung cancer.

### 3.2 Molecular characteristics

Driver gene variants including epidermal growth factor receptor (EGFR) mutations, anaplastic lymphoma kinase (ALK)/c‐ros oncogene 1 (ROS1)/rearranged during transfection fusion, v‐raf murine sarcoma viral oncogene homolog B1 (BRAF) V600E, and mesenchymal to epithelial transition factor (MET) amplification/exon 14 skipping mutations were more frequently found in non‐smokers than that in smokers in both 295 cohort and 1021 cohort (295 cohort, 30.08% (37/123) in smokers vs. 65.27% (105/161) in non‐smokers, *p* < 0.0001; 1021 cohort, 33.11% (49/148) in smokers vs. 73.33% (176/240) in non‐smokers, *p* < 0.0001) (Figure S1). However, KRAS and PIK3CA mutations were more common in smokers than that in non‐smokers (295 cohort, 40.65% (50/123) in smokers vs. 16.77% (27/161) in non‐smokers, *p* < 0.0001; 1,021 cohort, 33.11% (49/148) in smokers vs. 13.75% (33/240) in non‐smokers, *p* < 0.0001) (Figure S1). In the 295 cohort, 3.13% (1/32), 0.00% (0/32), and 0.00% (0/32) of participants who received ICI combined with chemotherapy or antiangiogenic therapy comprised EGFR, ALK, or ROS1 gene variants, respectively. In the 1021 cohort, 12.07% (7/58), 3.45% (2/58), and 0.00% (0/58) of participants who received ICI combined with chemotherapy or antiangiogenic therapy comprised EGFR, ALK, or ROS1 gene variants, respectively.

### 3.3 Correlation between TMB and the smoking status

We then compared TMB data across two cohorts. In the 295 cohort, the average TMB values in all participants (323 cases), lung adenocarcinoma (LUAD, 272 cases), lung squamous cell carcinoma (LUSC, 45 cases), and adenosquamous carcinoma (ASQC, 6 cases), were 8.787 ± 7.732, 8.271 ± 7.320, 10.860 ± 8.502, and 16.670 ± 13.470 mutations/Mb, respectively. In the 1021 cohort, the TMB values in all participants (388 cases), LUAD (328 cases), LUSC (50 cases), and ASQC (10 cases) were 7.169 ± 6.855, 6.631 ± 6.815, 10.500 ± 6.489, and 8.544 ± 5.732 mutations/Mb, respectively (Figure S2).

**FIGURE 1:**
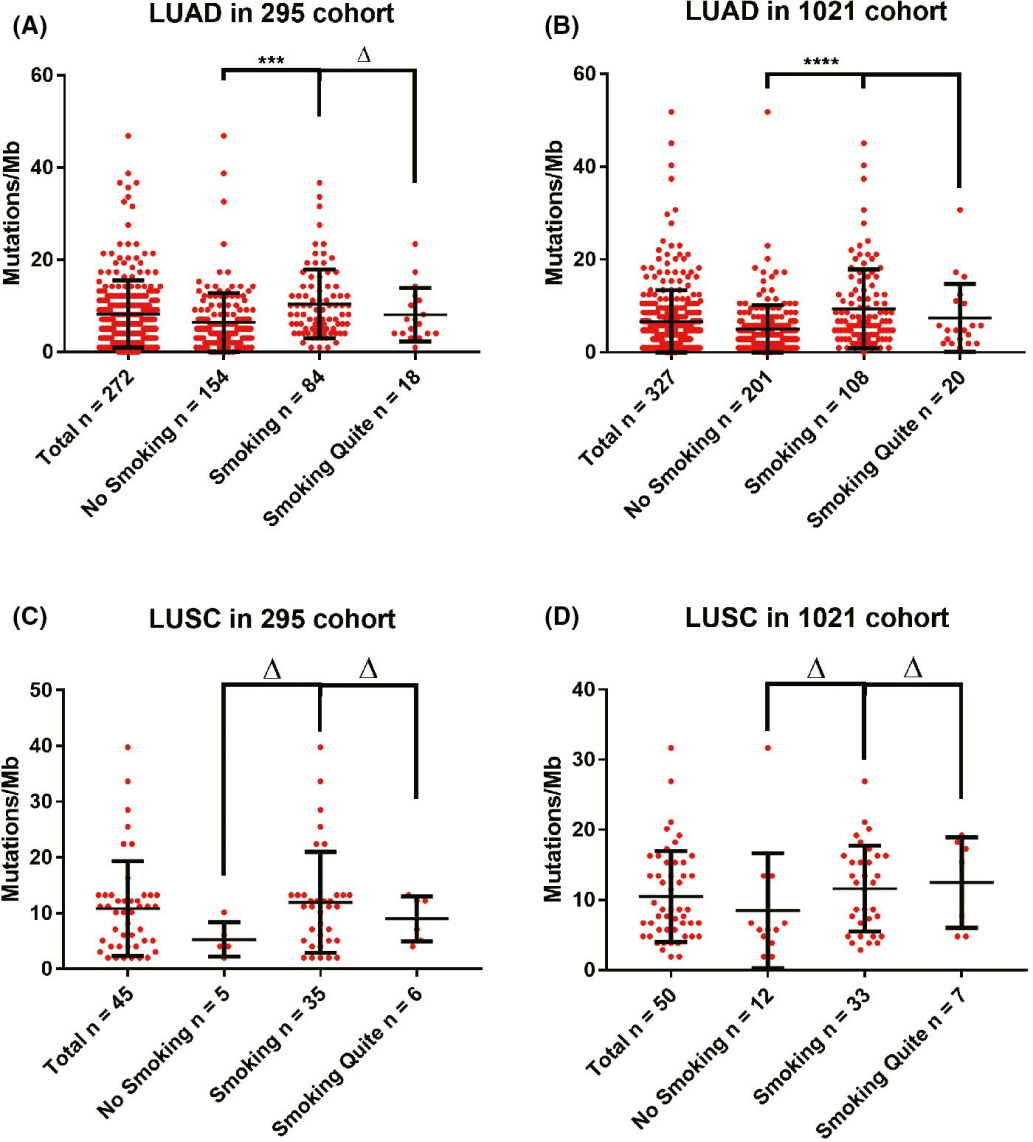
The smoking patients had higher TMB value than non‐smoking patients in LUAD. Plot showing TMB values of NSCLC patients with different smoking status in the 295 cohort (A, C) and the 1021 cohort (B, D). LUAD, lung adenocarcinoma; LUSC, lung squamous cell carcinoma; NSCLC, non‐small cell lung cancer; TMB, tumor mutational burden

**FIGURE 2:**
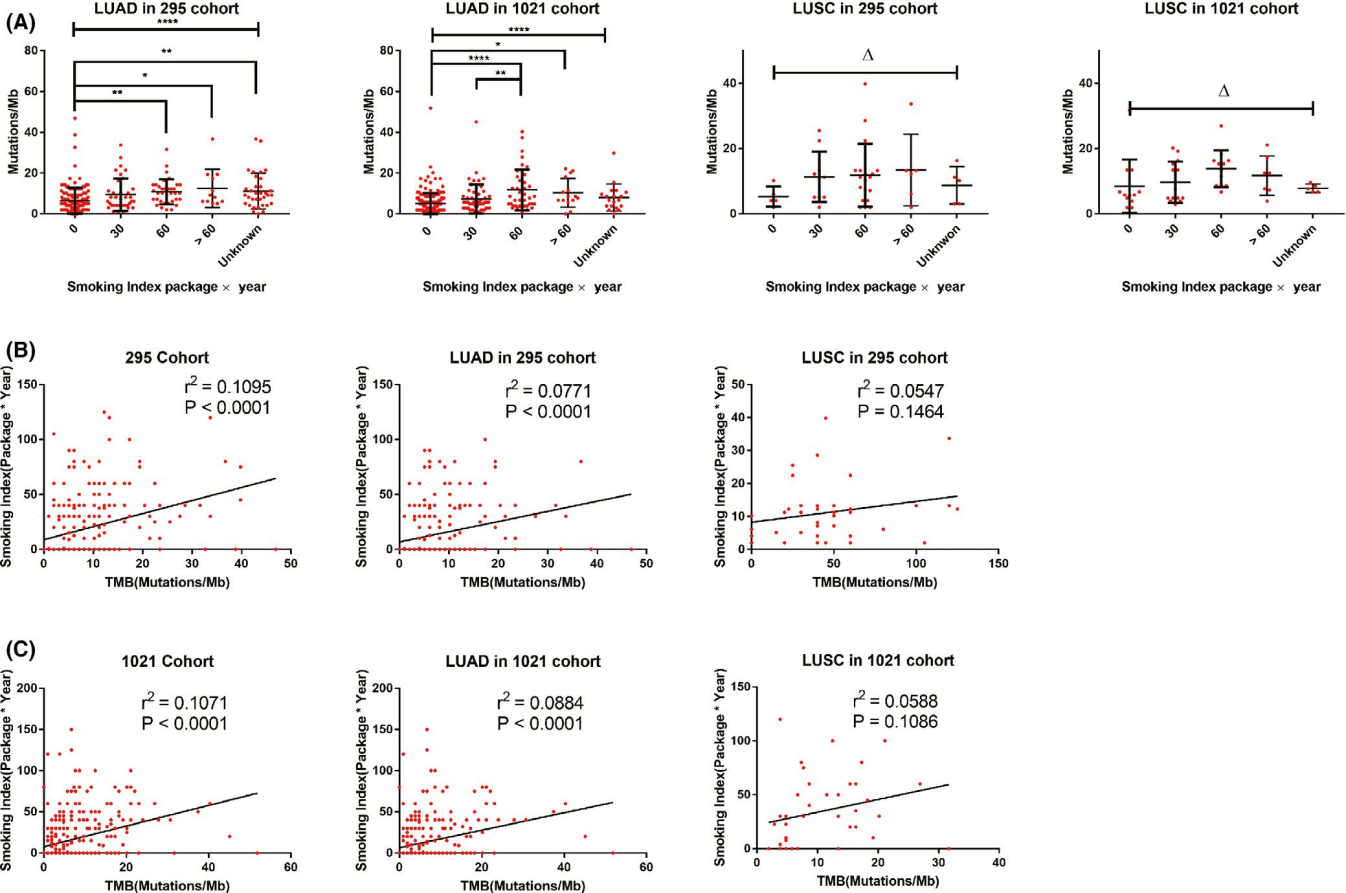
TMB values correlated with the smoking index in the 295 cohort and the 1021 cohort. LUAD, lung adenocarcinoma; LUSC, lung squamous cell carcinoma; NSCLC, non‐small cell lung cancer; TMB, tumor mutational burden

The TMB value in smoking LUAD patients was significantly higher than that in non‐smokers in both cohorts (295 cohort, 10.430 ± 7.468 mutations/Mb in 84 cases smoking patients vs. 6.452 ± 6.366 mutations/Mb in 154 cases non‐smoking patients, *p* = 0.0002; 1021 cohort, 9.374 ± 8.479 mutations/Mb in 108 cases smoking patients vs. 5.010 ± 5.048 mutations/Mb in 201 cases non‐smoking patients, *p* < 0.0001) (Figure 1A,B). However, there was no statistical TMB difference between smoking quiet and smoking in LUAD patients (295 cohort, 10.430 ± 7.468 mutations/Mb in 84 cases smoking patients vs. 8.105 ± 5.799 mutations/Mb in 18 cases smoking quiet patients, 10.430 ± 7.468 vs., *p* = 0.5796; 1021 cohort, 9.374 ± 8.479 mutations/Mb in 108 cases smoking patients vs. 7.418 ± 7.305 mutations/Mb in 20 cases smoking quiet patients, *p* = 0.6258) (Figure 1A,B). The TMB value of LUAD patients with a smoking index greater than 30 packs × years was significantly greater than that of non‐smoking patients (Figure 2). Although the TMB value of smoking LUSC patients in the two cohorts was higher than that in non‐smoking patients, the difference was not statistically significant (295 cohort, 11.950 ± 9.066 mutation/Mb in 35 cases smoking patients vs. 5.034 ± 3.094 mutations/Mb in 5 cases non‐smoking patients, *p* = 0.3503; 1021 cohort, 9.374 ± 8.479 mutations/Mb in 33 cases smoking patients vs. 5.010 ± 5.048 mutations/Mb in 12 cases non‐smoking patients, *p* = 0.2278) (Figure 1C,D).

Correlation analysis showed that in both 295 cohort and 1021 cohort, the smoking index and TMB were correlated (295 cohort, *r* ^2^ = 0.1095, *p* < 0.0001; 1021 cohort, *r* ^2^ = 0.1071, *p* < 0.0001). In LUAD, smoking index and TMB were correlated as well (295 cohort, *r* ^2^ = 0.0771, *p* < 0.0001; 1021 cohort, *r* ^2^ = 0.0884, *p* < 0.0001). However, in LUSC, the smoking index was not correlated with TMB (cohort 295, *r* ^2^ = 0.0547, *p* = 0.1464; cohort 1021, *r* ^2^ = 0.0588, *p* = 0.1086) (Figure 2).

### 3.4 Clinical outcomes

The median follow‐up time of the 295 cohort and the 1021 cohort was 6.47 and 6.17 months, respectively. In the 295 cohort, the PFS of smoking patients was slightly longer than that of non‐smoking patients, but the difference was not statistically significant (9.17 months in smoking patients vs. 6.00 months in non‐smoking patients, *p* = 0.1070). The PFS of patients with TMB high (TMB‐H) was similar to that of patients with TMB low (TMB‐L) (6.93 months in TMB‐H vs. 7.77 months in TMB‐L, *p* = 0.7030). In the 1021 cohort, the PFS of non‐smokers was slightly longer than that of smokers, but the difference was not statistically significant (9.03 months in smoking patients vs. 10.23 months in non‐smoking patients, *p* = 0.1240). Patients with TMB‐H had a slightly longer PFS than patients with TMB‐L, but the difference was not statistically significant as well (HR = 0.51 [0.22–1.19], *p* = 0.1714) (Figure S3).

**FIGURE 3:**
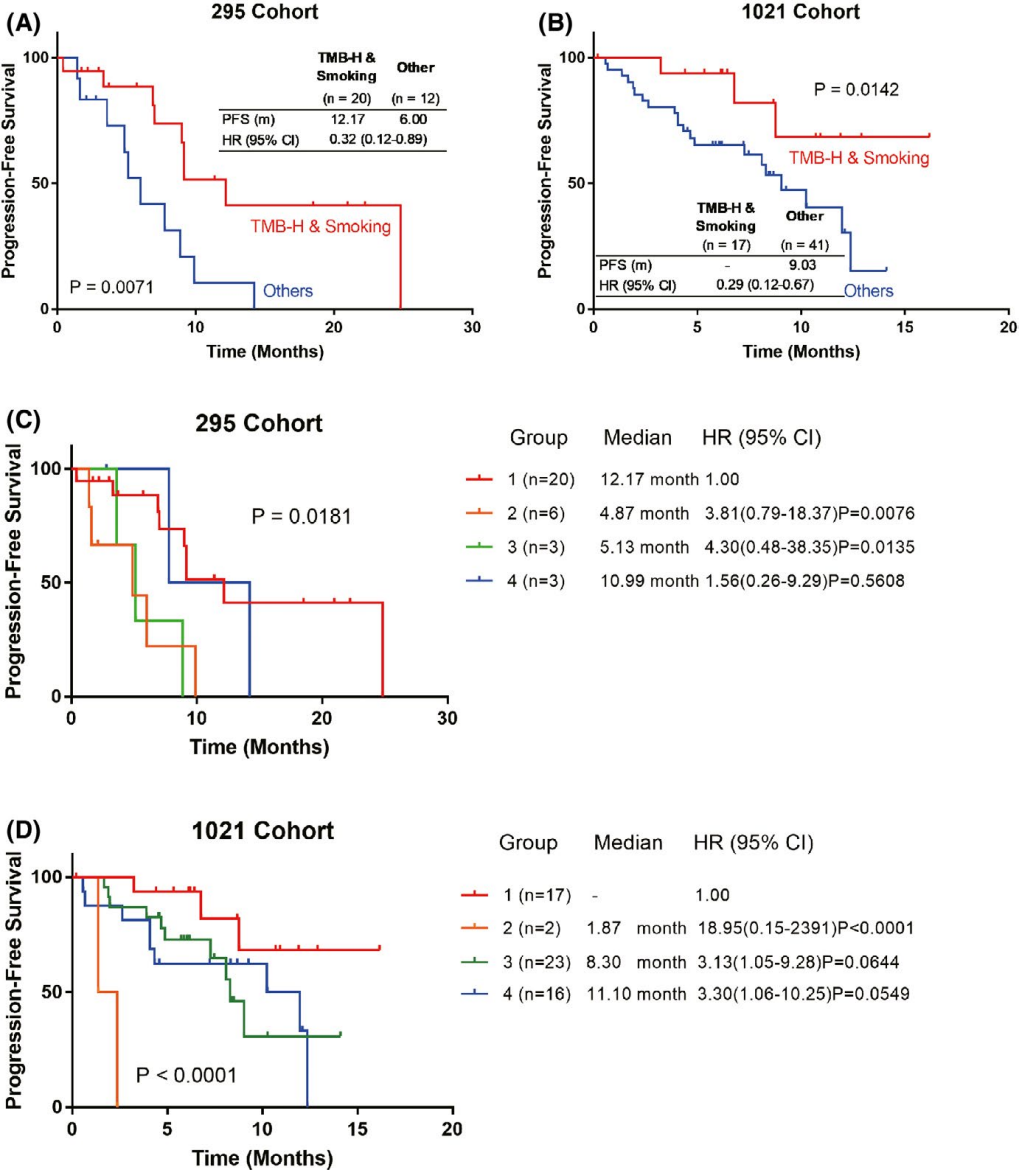
Kaplan–Meier survival curve showing the PFS of TMB high (TMB‐H)/smoking NSCLC patients and other patients in the 295 cohort and the 1021 cohort (A, B). Subgroup analysis showing the PFS of NSCLC patients with TMB‐H/smoking (Group 1), TMB‐H/non‐smoking (Group 2), TMB‐L/smoking (Group 3), and TMB‐L/non‐smoking (Group 4) in the 295 cohort and the 1021 cohort (C, D). NSCLC, non‐small cell lung cancer; PFS, progression‐free survival; TMB‐H, tumor mutational burden high; TMB‐L, tumor mutational burden Low

However, the PFS of patients with both TMB‐H and smoking status was significantly longer than other patients, and the difference was statistically significant in both cohorts (295 cohort, 12.17 months in both TMB‐H and smoking patients vs. 6.00 months in other patients, HR = 0.32 [0.12–0.89], *p* = 0.0071; 1021 cohort, HR = 0.29 [0.12–0.67], *p* = 0.0142) (Figure 3A,B). Further stratification analysis showed that patients with TMB‐H and no smoking had the shortest PFS in both cohorts (295 cohort, *p* = 0.0130; 1021 cohort, *p* < 0.0001) (Figure 3C,D).

## 4 DISCUSSION

In the clinical treatment of advanced NSCLC, ICI targeting PD1/PD‐L1 has made rapid progress,^17^ PD1/PD‐L1 inhibitor monotherapy or combination therapy with chemotherapy/antiangiogenic therapy has become one of the main treatment especially for NSCLC patients without the presence of driver gene mutation.^18^ However, currently there is no effective biomarkers for the efficacy prediction of combination immunotherapy, and the relationship between TMB and smoking status in NSCLC is controversial.^11^, ^19^ In this study, we retrospectively recruited 711 cases NSCLC patients who received two different NGS panel tests to analyze the relationship between TMB and smoking status, and we further evaluated the efficacy predictive value of smoking status and TMB in 90 cases NSCLC patients who received ICI therapy combined with chemotherapy/anti‐angiogenesis therapy.

Tumor mutational burden as a predictive marker of ICI efficacy is controversial in multiple studies, moreover, there is a lack of evidence for its application in ICI combination therapy.^4^, ^5^, ^6^, ^7^ Chen et al. found that TMB could not predict treatment efficacy in advanced NSCLC patients treated with camrelizumab combined with apatinib.^20^ Another study showed that TMB could not predict the efficacy of sintilimab combined with dual‐drug chemotherapy.^21^ In addition, the definition of high TMB measured by different NGS detection platforms (different gene panels) might be different.^22^ Our results also found in the two different patients cohorts that TMB alone could not predict the prognosis of advanced NSCLC patients receiving ICI combination therapy in both cohorts.

Smoking NSCLC patients may carry more gene mutations, especially TP53, KRAS, and PIK3.^19^, ^23^ Therefore, the TMB value in smoking patients is supposed to be increased.^24^ However, the correlation between the smoking index and the TMB value of NSCLC patients is still controversial.^11^ Previous studies have shown that smoking patients receiving ICI monotherapy have better response rates and PFS than non‐smokers, but there is no difference in overall survival between two groups.^25^ In ICI combined chemotherapy/anti‐angiogenesis therapy, it has also been shown that both smokers and non‐smokers could benefit from combination therapy compared to chemotherapy alone.^26^, ^27^ Another study also showed that in patients with PD‐L1 expression ≥50%, non‐smokers had shorter PFS than heavy‐smokers, but the difference was not statistically significant.^12^ Our study also found that TP53, KRAS, and PIK3 gene mutations in smoking patients were increased compared with non‐smoking patients. In the two cohorts, the TMB value in LUAD was correlated with the smoking index (295 cohort, *r* ^2^ = 0.0771, *p* < 0.0001; 1021 cohort, *r* ^2^ = 0.0884, *p* < 0.0001). However, the TMB value of LUSC has nothing to do with the smoking index. Our results also found that smoking status alone cannot predict the prognosis of NSCLC patients treated with ICI combination therapy. Taken together, our results showed that neither TMB alone nor smoking status alone could predict the efficacy of ICI combination therapy in advanced NSCLC.

Next, we speculated that combining the two indicators may predict the efficacy of ICI combination therapy. After combining TMB value with smoking status, we found that TMB‐H/smoking patients had much better PFS than other patients in both 295 cohort and 1021 cohort, suggesting that TMB combined with smoking status might be an efficient predictor of NSCLC patients receiving ICI combination therapy. Meanwhile the PFS of TMB‐H/non‐smoking patients was the shortest in both cohorts, suggesting that these patients may have different resistance mechanisms of ICI combination therapy.

Interestingly, the PFS of TMB‐L/non‐smoking patients and TMB‐H/smoking patients was quite similar, and the difference was not statistically significant (295 cohort, *p* = 0.5608; 1021 cohort, *p* = 0.0549). Previous study has found that patients with low TMB have better efficacy than those with moderate TMB after receiving ICI treatment.^28^ This suggests that the role of TMB in predicting the efficacy of ICI may not be a purely linear relationship, and further research is needed to elucidate its role in ICI combination therapy.

Although our study found that TMB combined with smoking status was a potential predictor of efficacy in advanced NSCLC patients receiving ICI combination therapy, the study had the following shortcomings: (1) The sample size in this study was small, which may lead to bias; (2) About 10% of patients had driver gene mutations; and 3. Different ICI (different PD‐1 antibodies or PD‐L1 antibodies) and combination therapy (chemotherapy and/or anti‐angiogenesis therapy) included in this study, and the potential different effects of such drugs cannot be ruled out.

## 5 CONCLUSIONS

This study retrospectively analyzed advanced NSCLC patients receiving ICI combined with chemotherapy/anti‐angiogenesis therapy in two different NGS detection cohorts. The TMB value correlated with smoking status in LUAD patients, but not in LUSC patients. The results showed that TMB value combined with smoking status could be used as a potential prognostic indicator for advanced NSCLC patients receiving ICI combination therapy. This study provided a potential prognostic indicator for the personalized immunotherapy of advanced NSCLC.

## AUTHOR CONTRIBUTIONS

Conceptualization, Li‐Yue Sun, Zi‐Ming Du, and Jian‐Yong Shao; methodology, Li‐Yue Sun, Zi‐Ming Du, and Xin‐Hua Yang.; validation, Li‐Yue Sun, Wen‐Jian Cen, Wen‐Ting Tang, and Ya‐Kong Long; formal analysis, Xin‐Hua Yang and Xiao‐Meng Ji; investigation, Li‐Yue Sun and Zi‐Ming Du; resources, Fang Wang and Jian‐Yong Shao; data curation, Wen‐Jian Cen, Wen‐Ting Tang, Jiao‐Jiao Yang, and Ren‐Jing Zhang; writing—original draft preparation, Li‐Yue Sun; writing—review and editing, Zi‐Ming Du; supervision, Jian‐Yong Shao; project administration, Zi‐Ming Du; All authors have read and agreed to the published version of the manuscript.

## CONFLICTS OF INTEREST

The authors declare that they have no conflicts of interest.

## ETHICAL STATEMENT

The authors are accountable for all aspects of the work in ensuring that questions related to the accuracy or integrity of any part of the work are appropriately investigated and resolved. The present study was approved by the Clinical Research Ethics Committee of Sun Yat‐sen University Cancer Center (no. B2020‐344–01). All procedures were strictly in accordance with the appropriate version of the Declaration of Helsinki (as revised in Brazil 2013). Individual consent for this retrospective analysis was waived.

## Supporting information

Supplementary Material

## Data Availability

The data that support the findings of this study are available from the corresponding author upon reasonable request.
