# Supplementary material for: Smoking status combined with tumor mutational burden as a prognosis predictor for combination immune checkpoint inhibitor therapy in non‐small cell lung cancer
Source: Cancer Med. 2021 Sep 1;10(19):6610–7. doi: 10.1002/cam4.4197 (PMC8495280; doi:10.1002/cam4.4197)
Supplement: Supplementary file 1 — Supplementary Material [file CAM4-10-6610-s001.docx]

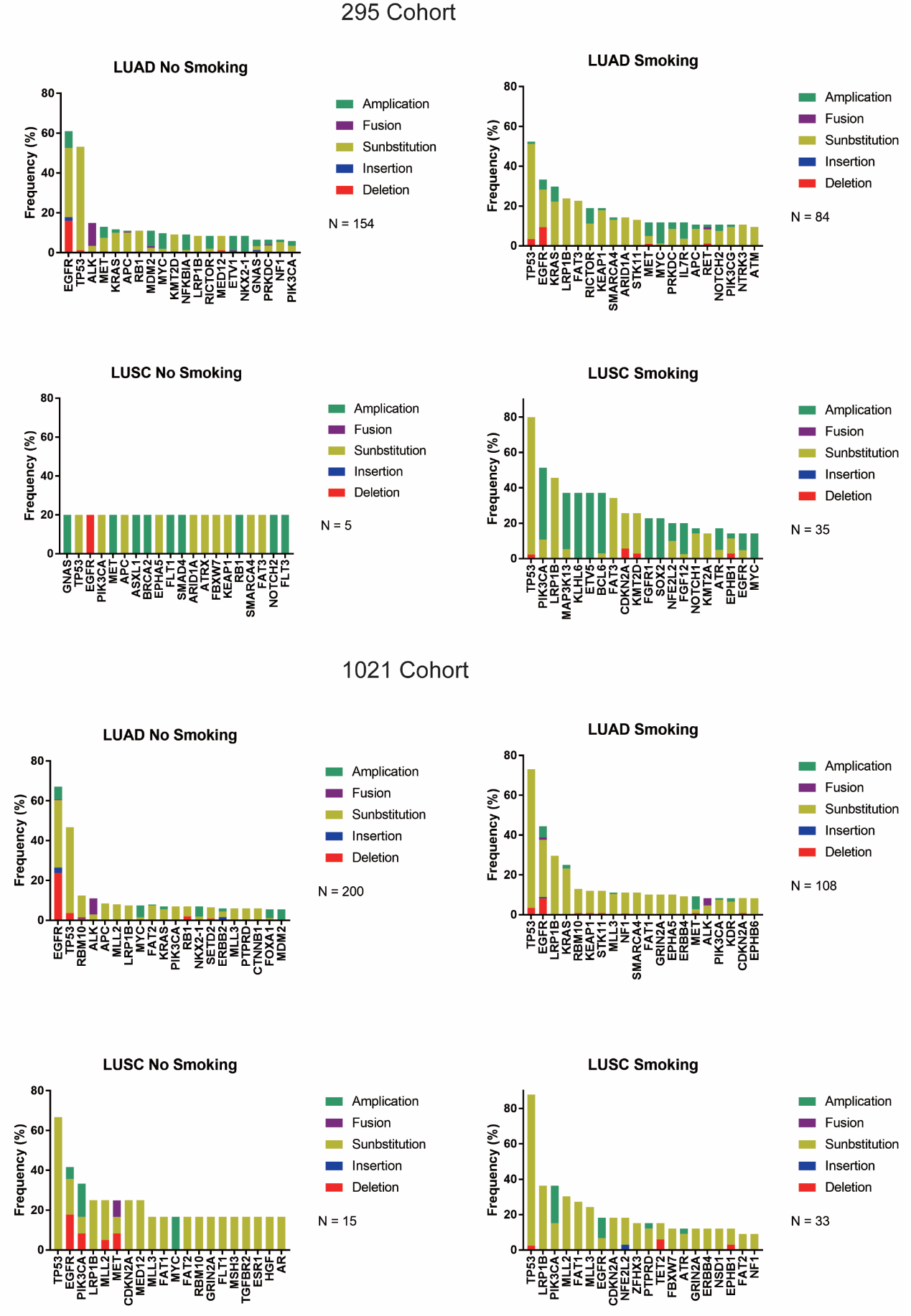


**Supplementary Figure 1.** Gene mutation profile in NSCLC patients in two cohorts;

Abbreviations: LUAD, lung adenocarcinoma; LUSC, lung squamous cell carcinoma;


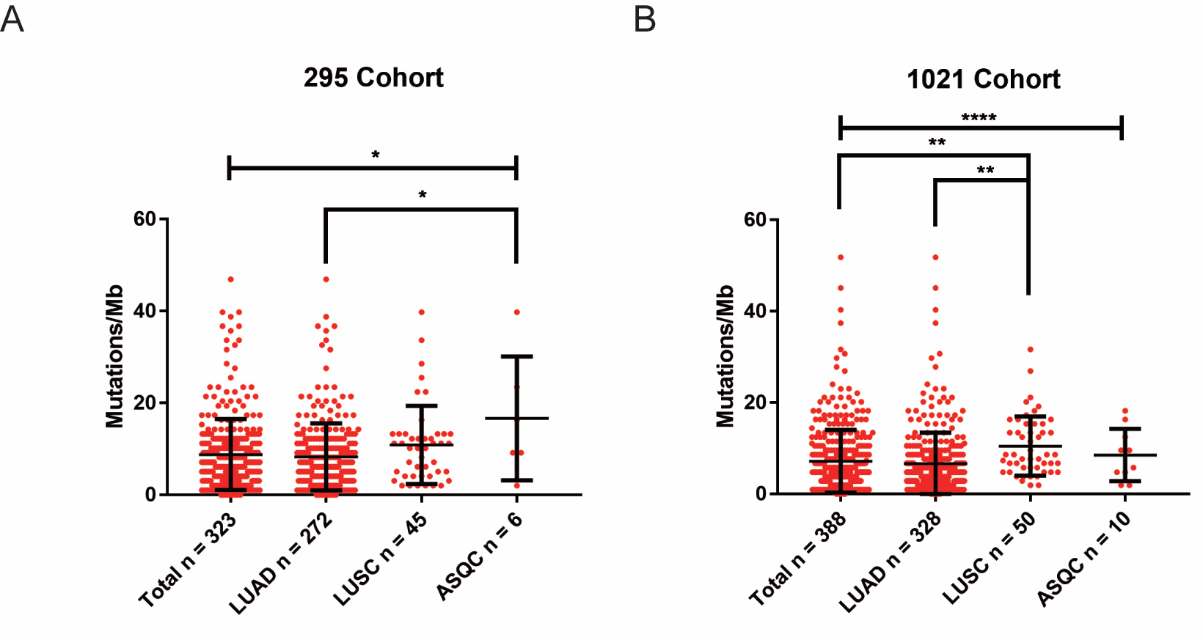


**Supplementary Figure 2.** Dot plots showing TMB value of total NSCLC, LUAD, LUSC, and ASQC patients in the 295 cohort **(A)** and the 1021 cohort **(B).**

Abbreviations: TMB, tumor mutational burden; NSCLC, non-small cell lung; LUAD, lung adenocarcinoma; LUSC, lung squamous cell carcinoma; ASQC, adenosquamous carcinoma.


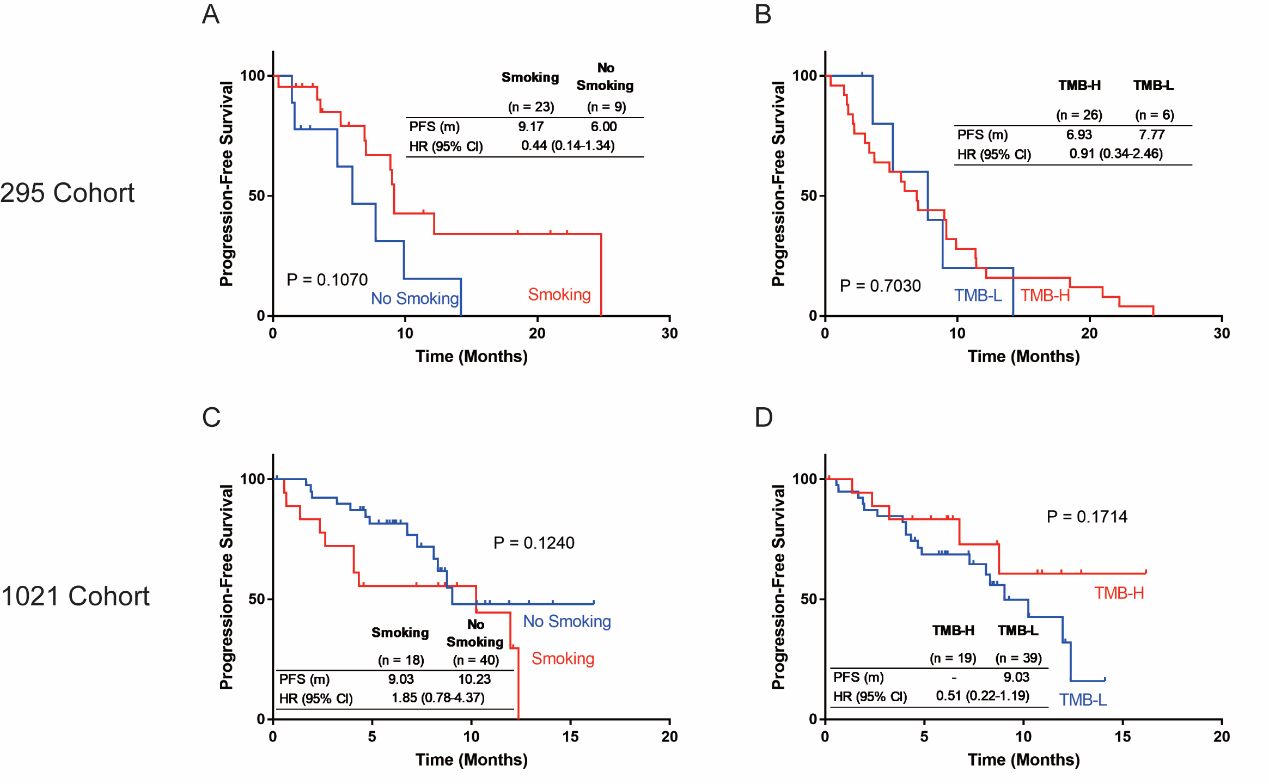


**Supplementary Figure 3.** Kaplan-Meier survival curve showing the PFS of NSCLC patients with or without smoking, with TMB High (TMB-H) or with TMB Low (TMB-L) in the 295 cohort and the 1021 cohort.

Abbreviations: PFS, progression-free survival; TMB-H, tumor mutational burden high; TMB-L, tumor mutational burden Low; NSCLC, non-small cell lung cancer.

**Supplementary Table 1.** Clinical characteristics of NSCLC patients receiving combination therapy

| Characteristic |  | 295 Cohort | 1021 Cohort | P value |
| --- | --- | --- | --- | --- |
| Number |  | 32 | 58 |  |
| Age |  | 59.91±10.55 | 59.84±10.42 | 0.842 |
| Gender, n (%) |  |  |  | 0.569 |
|  | Male | 28(87.5) | 50(86.2) |  |
|  | Female | 4(12.5) | 8(13.8) |  |
| Histology, n (%) |  |  |  | 0.995 |
|  | Adenocarcinoma | 19(59.4) | 34(58.6) |  |
|  | Squamous cell | 12(37.5) | 22(37.9) |  |
|  | Adenosquamous | 1(3.1) | 2(3.4) |  |
| TNM Stage, n (%) | |  |  | **0.000** |
|  | II | 1(3.1) | 0(0.0) |  |
|  | III | 3(9.4) | 29(50.0) |  |
|  | IV | 28(87.5) | 29(50.0) |  |
| Smoking statue |  |  |  | 0.929 |
|  | Current | 19(59.4) | 34(58.6) |  |
|  | Never | 9(28.1) | 18(31.0) |  |
|  | Former | 4(12.5) | 6(10.3) |  |
| Smoking index (page × year) | | 34.22±28.94 | 32.16±33.42 | 0.265 |
| TMB (Muts/Mb) |  | 12.40±9.33 | 12.58±10.08 | 0.546 |
| Drive gene variants (EGFR, ALK, ROS1, BRAF and MET) | | | | 0.245 |
|  | Yes | 3(9.4) | 10(17.2) |  |
|  | No | 29(90.6) | 48(82.8) |  |
| KRAS and PIK3 gene variants | |  |  | 0.361 |
|  | Yes | 13(40.6) | 20(34.5) |  |
|  | No | 19(59.4) | 38(65.5) |  |
| Prior lines of therapy, n (%) | |  |  | 0.953 |
|  | 0 | 22(68.8) | 38(65.5) |  |
|  | 1 | 6(18.8) | 12(20.7) |  |
|  | ≥2 | 4(12.5) | 8(13.8) |  |
| PD1/PD-L1 inhibitor | |  |  | **0.008** |
|  | Atezolizumab | 0(0.0) | 1(1.7) |  |
|  | Durvalumab | 0(0.0) | 1(1.7) |  |
|  | Camrelizumab | 2(6.2) | 4(6.9) |  |
|  | Nivolumab | 4(12.5) | 27(46.6) |  |
|  | Pembrolizumab | 17(53.1) | 13(22.4) |  |
|  | Toripalimab | 6(18.8) | 3(5.2) |  |
|  | Tislelizumab | 0(0.0) | 2(3.4) |  |
|  | Sintilimab | 3(9.4) | 7(12.1) |  |
| Abbreviations: TMB, tumor mutation burden; | | | | |

| **Supplementary Table 2**. Gene list of 295-gene panel | | | | | | | | | | |  | |  |
| --- | --- | --- | --- | --- | --- | --- | --- | --- | --- | --- | --- | --- | --- |
| ABL1 | AKT1 | | AKT2 | | AKT3 | | ALK | | ALOX12B | | AMER1 | |  |
| APC | APCDD1 | | AR | | ARAF | | ARFRP1 | | ARID1A | | ARID2 | |  |
| ASXL1 | ATM | | ATR | | ATRX | | AURKA | | AURKB | | AXL | |  |
| BACH1 | BAP1 | | BARD1 | | BCL2 | | BCL2L2 | | BCL6 | | BCOR | |  |
| BCORL1 | BCR | | BLM | | BRAF | | BRCA1 | | BRCA2 | | BRIP1 | |  |
| BTG1 | BTK | | C11ORF30 | | CARD11 | | CASP8 | | CBFB | | CBL | |  |
| CCND1 | CCND2 | | CCND3 | | CCNE1 | | CD79A | | CD79B | | CDC73 | |  |
| CDH1 | CDK12 | | CDK4 | | CDK6 | | CDK8 | | CDKN1B | | CDKN2A | |  |
| CDKN2B | CDKN2C | | CEBPA | | CHEK1 | | CHEK2 | | CHUK | | CIC | |  |
| CRBN | CREBBP | | CRKL | | CRLF2 | | CSF1R | | CTCF | | CTNNA1 | |  |
| CTNNB1 | CUL4A | | CUL4B | | CYP17A1 | | DAXX | | DDR2 | | DIS3 | |  |
| DNMT3A | DOT1L | | EGFR | | EP300 | | EPHA3 | | EPHA5 | | EPHB1 | |  |
| ERBB2 | ERBB3 | | ERBB4 | | ERG | | ESR1 | | ETV1 | | ETV4 | |  |
| ETV5 | ETV6 | | EWSR1 | | EZH2 | | FAM46C | | FANCA | | FANCC | |  |
| FANCD2 | FANCE | | FANCF | | FANCG | | FANCI | | FANCL | | FANCM | |  |
| FAT3 | FBXW7 | | FGF10 | | FGF12 | | FGF14 | | FGF19 | | FGF23 | |  |
| FGF3 | FGF4 | | FGF6 | | FGF7 | | FGFR1 | | FGFR2 | | FGFR3 | |  |
| FGFR4 | FLT1 | | FLT3 | | FLT4 | | FOXL2 | | GATA1 | | GATA2 | |  |
| GATA3 | GID4 | | GNA11 | | GNA13 | | GNAQ | | GNAS | | GPR124 | |  |
| GRIN2A | GSK3B | | HGF | | HLA-A | | HRAS | | IDH1 | | IDH2 | |  |
| IGF1 | IGF1R | | IGF2 | | IKBKE | | IKZF1 | | IL7R | | INHBA | |  |
| IRF4 | IRS2 | | JAK1 | | JAK2 | | JAK3 | | JUN | | KAT6A | |  |
| KDM5A | KDM5C | | KDM6A | | KDR | | KEAP1 | | KIT | | KLHL6 | |  |
| KMT2A | KMT2D | | KRAS | | LMO1 | | LRP1B | | MAP2K1 | | MAP2K2 | |  |
| MAP2K4 | MAP3K1 | | MAP3K13 | | MCL1 | | MDM2 | | MDM4 | | MED12 | |  |
| MEF2B | MEN1 | | MET | | MITF | | MLH1 | | MPL | | MRE11A | |  |
| MSH2 | MSH6 | | MTOR | | MUTYH | | MYC | | MYCL1 | | MYCN | |  |
| MYD88 | NBN | | NCOR1 | | NF1 | | NF2 | | NFE2L2 | | NFKBIA | |  |
| NKX2-1 | NOTCH1 | | NOTCH2 | | NOTCH3 | | NOTCH4 | | NPM1 | | NRAS | |  |
| NSD1 | NTRK1 | | NTRK2 | | NTRK3 | | NUP93 | | PAK3 | | PAK7 | |  |
| PALB2 | PARP1 | | PARP2 | | PARP3 | | PARP4 | | PAX5 | | PBRM1 | |  |
| PDGFRA | PDGFRB | | PDK1 | | PIK3C2G | | PIK3C3 | | PIK3CA | | PIK3CG | |  |
| PIK3R1 | PIK3R2 | | PMS2 | | PNRC1 | | PPP2R1A | | PRDM1 | | PRKAR1A | |  |
| PRKDC | PRSS8 | | PTCH1 | | PTEN | | PTPN11 | | RAD50 | | RAD51 | |  |
| RAD51B | RAD51C | | RAD51D | | RAD52 | | RAD54L | | RAF1 | | RARA | |  |
| RB1 | REL | | RET | | RICTOR | | RNF43 | | RPA1 | | RPTOR | |  |
| ROS1 | RUNX1 | | RUNX1T1 | | SETD2 | | SF3B1 | | SH2B3 | | SMAD2 | |  |
| SMAD4 | SMARCA4 | | SMARCB1 | | SMARCD1 | | SMO | | SOCS1 | | SOX10 | |  |
| SOX2 | SPEN | | SPOP | | SRC | | STAG2 | | STAT4 | | STK11 | |  |
| SUFU | SYK | | TBX3 | | TET2 | | TGFBR2 | | TIPARP | | TMPRSS2 | |  |
| TNFAIP3 | TNFRSF14 | | TOP1 | | TP53 | | TRRAP | | TSC1 | | TSC2 | |  |
| TSHR | VHL | | WISP3 | | WT1 | | XPO1 | | XRCC3 | | ZNF217 | |  |
| ZNF703 |  | |  | |  | |  | |  | |  | |  |
| **Supplementary Table 3.** Gene list of 1021-gene panel | | | | | | | | | | | | | |
| ABCA13 | | ABCB1 | | ABCC1 | | ABCC11 | | ABCC2 | | ABCG2 | | ABL1 | |
| ABL2 | | ACACA | | ACIN1 | | ACTB | | ACTG1 | | ACTG2 | | ACVR1B | |
| ACVR2A | | ACVRL1 | | ADAM29 | | ADAMTS5 | | ADCY1 | | AFF1 | | AFF2 | |
| AFF3 | | AHNAK | | AKAP9 | | AKT1 | | AKT2 | | AKT3 | | ALB | |
| ALK | | AMOT | | ANGPT1 | | ANK3 | | ANKRD11 | | ANKRD30A | | ANKRD30B | |
| APC | | APEX1 | | APOBEC3B | | AR | | ARAF | | ARAP3 | | ARFGEF1 | |
| ARFGEF2 | | ARHGAP29 | | ARHGAP35 | | ARID1A | | ARID1B | | ARID2 | | ARID4B | |
| ARID5B | | ARNT | | ASCL4 | | ASH1L | | ASMTL | | ASPM | | ASTN1 | |
| ASXL1 | | ASXL2 | | ATIC | | ATM | | ATP11B | | ATP12A | | ATP1A1 | |
| ATP2B3 | | ATR | | ATRX | | AURKA | | AURKB | | AXIN1 | | AXIN2 | |
| AXL | | B2M | | BAP1 | | BARD1 | | BAZ2B | | BBC3 | | BBS9 | |
| BCAS1 | | BCL10 | | BCL11A | | BCL11B | | BCL2 | | BCL2A1 | | BCL2L1 | |
| BCL2L11 | | BCL3 | | BCL6 | | BCL9 | | BCOR | | BCORL1 | | BCR | |
| BIRC3 | | BLM | | BMPR1A | | BMPR2 | | BNC2 | | BPTF | | BRAF | |
| BRCA1 | | BRCA2 | | BRD2 | | BRD3 | | BRD4 | | BRIP1 | | BRSK1 | |
| BRWD1 | | BTK | | BTLA | | BUB1 | | C11orf30 | | C15orf23 | | C15orf55 | |
| C1QA | | C1S | | C3orf70 | | C7orf53 | | C8orf34 | | CACNA1E | | CADM2 | |
| CALR | | CAMTA1 | | CARD11 | | CASP1 | | CASP8 | | CASQ2 | | CBFB | |
| CBL | | CBLB | | CBR1 | | CBR3 | | CCDC168 | | CCNA1 | | CCNB3 | |
| CCND1 | | CCND2 | | CCND3 | | CCNE1 | | CCT3 | | CCT5 | | CCT6B | |
| CD22 | | CD274 | | CD33 | | CD5L | | CD74 | | CDA | | CDC73 | |
| CDH1 | | CDH11 | | CDH18 | | CDH23 | | CDK12 | | CDK13 | | CDK4 | |
| CDK6 | | CDK8 | | CDKN1A | | CDKN1B | | CDKN2A | | CDKN2B | | CDKN2C | |
| CEBPA | | CHD1 | | CHD1L | | CHD4 | | CHD6 | | CHD8 | | CHD9 | |
| CHEK1 | | CHEK2 | | CHFR | | CHI3L1 | | CHN1 | | CIC | | CIITA | |
| CLDN18 | | CLP1 | | CLSPN | | CLTC | | CNOT3 | | CNOT4 | | CNTN1 | |
| CNTN5 | | CNTNAP1 | | CNTNAP5 | | COL1A1 | | COL2A1 | | COL5A1 | | COL5A2 | |
| COL5A3 | | COPS2 | | CPS1 | | CREBBP | | CRIPAK | | CRKL | | CRLF2 | |
| CRNKL1 | | CRTC1 | | CSF1 | | CSF1R | | CSF3R | | CSMD1 | | CSMD3 | |
| CSNK1A1 | | CSNK1G3 | | CTCF | | CTLA4 | | CTNNA1 | | CTNNA2 | | CTNNB1 | |
| CTNND1 | | CUL3 | | CUX1 | | CXCR4 | | CYBA | | CYLD | | CYP19A1 | |
| CYP1A1 | | CYP1B1 | | CYP2A13 | | CYP2C8 | | CYP2D6 | | CYP3A4 | | CYP3A5 | |
| DAXX | | DCC | | DDR1 | | DDR2 | | DDX3X | | DDX5 | | DEK | |
| DHX35 | | DHX9 | | DIAPH1 | | DICER1 | | DIS3L2 | | DLC1 | | DMD | |
| DNAH6 | | DNAJB1 | | DNM2 | | DNMT1 | | DNMT3A | | DNMT3B | | DOCK2 | |
| DOCK7 | | DOT1L | | DPYD | | DRGX | | DTX1 | | DUSP22 | | DYSF | |
| E2F3 | | EBF1 | | ECT2L | | EED | | EEF1A1 | | EGFL7 | | EGFR | |
| EGR3 | | EIF1AX | | EIF2AK3 | | EIF2C3 | | EIF3A | | EIF4A2 | | EIF4G3 | |
| ELAC2 | | ELF1 | | ELF3 | | ELMO1 | | ELN | | EME2 | | EMID2 | |
| EML4 | | EP300 | | EPAS1 | | EPC1 | | EPCAM | | EPHA1 | | EPHA2 | |
| EPHA3 | | EPHA4 | | EPHA5 | | EPHA7 | | EPHB1 | | EPHB2 | | EPHB4 | |
| EPHB6 | | EPOR | | EPPK1 | | EPS15 | | ERBB2 | | ERBB2IP | | ERBB3 | |
| ERBB4 | | ERCC1 | | ERCC2 | | ERCC3 | | ERCC4 | | ERCC5 | | ERG | |
| ERRFI1 | | ESR1 | | ESR2 | | ETS1 | | ETV1 | | ETV5 | | ETV6 | |
| EWSR1 | | EXT1 | | EXT2 | | EZH2 | | EZR | | F8 | | FAM123B | |
| FAM131B | | FAM135B | | FAM157B | | FAM175A | | FAM46C | | FAM5C | | FANCA | |
| FANCC | | FANCD2 | | FANCE | | FANCF | | FANCG | | FANCL | | FANCM | |
| FAP | | FAS | | FASLG | | FAT1 | | FAT2 | | FAT3 | | FAT4 | |
| FBXW7 | | FCGR1A | | FCGR2A | | FCGR2B | | FCGR3A | | FCRL4 | | FGF10 | |
| FGF12 | | FGF14 | | FGF19 | | FGF23 | | FGF3 | | FGF4 | | FGF6 | |
| FGFR1 | | FGFR2 | | FGFR3 | | FGFR4 | | FH | | FLCN | | FLG | |
| FLI1 | | FLNC | | FLT1 | | FLT3 | | FLT4 | | FMN2 | | FN1 | |
| FNDC4 | | FOXA1 | | FOXA2 | | FOXL2 | | FOXO1 | | FOXO3 | | FOXP1 | |
| FOXQ1 | | FRMPD4 | | FUBP1 | | FUS | | FXR1 | | FYN | | FZD1 | |
| G3BP1 | | G3BP2 | | GAB2 | | GABRA6 | | GALNT12 | | GATA1 | | GATA2 | |
| GATA3 | | GFRAL | | GIGYF1 | | GKN2 | | GLB1L3 | | GLI1 | | GLI2 | |
| GLI3 | | GMPS | | GNA11 | | GNA13 | | GNAQ | | GNAS | | GNG2 | |
| GPC3 | | GPR124 | | GPS2 | | GPX1 | | GRB7 | | GRIN2A | | GRM3 | |
| GSK3B | | GSTM5 | | GSTP1 | | GUSB | | H3F3A | | H3F3B | | H3F3C | |
| HCLS1 | | HCN1 | | HDAC1 | | HDAC4 | | HDAC9 | | HECW1 | | HEY1 | |
| HGF | | HIST1H1C | | HIST1H1D | | HIST1H1E | | HIST1H2AC | | HIST1H2AG | | HIST1H2AL | |
| HIST1H2AM | | HIST1H2BC | | HIST1H2BD | | HIST1H2BJ | | HIST1H2BK | | HIST1H2BO | | HIST1H3B | |
| HIST1H3C | | HIST1H3D | | HIST1H3F | | HIST1H3G | | HIST1H3H | | HIST1H3I | | HIST1H4I | |
| HIST3H3 | | HLA-A | | HLA-B | | HLA-C | | HLF | | HMCN1 | | HNF1A | |
| HNF1B | | HNRPDL | | HOXA11 | | HOXA13 | | HOXA3 | | HOXA9 | | HOXB13 | |
| HOXC13 | | HOXD11 | | HOXD13 | | HRAS | | HSD3B1 | | HSP90AA1 | | HSP90AB1 | |
| HSPA8 | | HSPD1 | | HSPH1 | | ICK | | ICOSLG | | ID3 | | IDH1 | |
| IDH2 | | IFITM3 | | IFNG | | IFNGR1 | | IGF1 | | IGF1R | | IGF2 | |
| IGF2R | | IGLL5 | | IKBKE | | IKZF1 | | IKZF2 | | IKZF3 | | IL10 | |
| IL1RAPL1 | | IL21R | | IL6 | | IL6ST | | IL7R | | IMPG1 | | ING1 | |
| INHBA | | INPP4A | | INPP4B | | INPPL1 | | INSR | | IRF2 | | IRF4 | |
| IRF6 | | IRS1 | | IRS2 | | ITGB3 | | ITK | | ITSN1 | | JAK1 | |
| JAK2 | | JAK3 | | JARID2 | | JUN | | KALRN | | KAT6A | | KAT6B | |
| KCNJ5 | | KCNQ2 | | KDM2B | | KDM5A | | KDM5C | | KDM6A | | KDR | |
| KEAP1 | | KEL | | KIF5B | | KIT | | KLF4 | | KLHL6 | | KLK1 | |
| KRAS | | KRTAP5-5 | | L3MBTL1 | | LAMA2 | | LATS1 | | LATS2 | | LCP1 | |
| LEF1 | | LGALS8 | | LIFR | | LPHN2 | | LPP | | LRP1B | | LRP2 | |
| LRP4 | | LRP5 | | LRP6 | | LRRC7 | | LRRK2 | | LYN | | LZTS1 | |
| MACF1 | | MAD1L1 | | MAF | | MAGI2 | | MAML2 | | MAML3 | | MAP2K1 | |
| MAP2K2 | | MAP2K4 | | MAP3K1 | | MAP3K13 | | MAPK1 | | MAPK3 | | MAX | |
| MCC | | MCL1 | | MCM3 | | MDC1 | | MDM2 | | MDM4 | | MECOM | |
| MED12 | | MEF2B | | MEF2C | | MEN1 | | MET | | MGA | | MIB1 | |
| MIOS | | MITF | | MKL1 | | MLH1 | | MLH3 | | MLL | | MLL2 | |
| MLL3 | | MLL4 | | MLLT3 | | MMP11 | | MMP2 | | MN1 | | MNDA | |
| MNX1 | | MPL | | MRE11A | | MS4A1 | | MSH2 | | MSH3 | | MSH4 | |
| MSH6 | | MSN | | MSR1 | | MST1R | | MTHFR | | MTOR | | MTRR | |
| MUC5B | | MUTYH | | MYC | | MYCL1 | | MYCN | | MYD88 | | MYH11 | |
| MYH14 | | MYH9 | | MYO3A | | MYOD1 | | NAP1L1 | | NAV3 | | NBN | |
| NCAM2 | | NCF2 | | NCF4 | | NCK1 | | NCOA3 | | NCOA4 | | NCOR1 | |
| NCOR2 | | NCSTN | | NDUFA13 | | NF1 | | NF2 | | NFATC4 | | NFE2L2 | |
| NFE2L3 | | NFKBIA | | NKX2-1 | | NKX3-1 | | NLRC3 | | NOD1 | | NOS3 | |
| NOTCH1 | | NOTCH2 | | NOTCH3 | | NOTCH4 | | NPM1 | | NQO1 | | NR1I2 | |
| NR2F2 | | NR4A2 | | NRAS | | NRG1 | | NRP2 | | NRXN1 | | NSD1 | |
| NTHL1 | | NTM | | NTRK1 | | NTRK2 | | NTRK3 | | NUMA1 | | NUP107 | |
| NUP210 | | NUP93 | | NUP98 | | OBSCN | | OGDH | | OMD | | OPCML | |
| OR11G2 | | OR2T4 | | OR4A15 | | OR4C6 | | OR5L2 | | OR6F1 | | P2RY8 | |
| P4HB | | PABPC1 | | PABPC3 | | PAG1 | | PAK1 | | PAK3 | | PALB2 | |
| PARK2 | | PARP1 | | PASK | | PAX3 | | PAX5 | | PAX7 | | PBRM1 | |
| PC | | PCDH18 | | PCK1 | | PCSK6 | | PCSK7 | | PDCD1 | | PDCD11 | |
| PDCD1LG2 | | PDE4DIP | | PDGFB | | PDGFRA | | PDGFRB | | PDILT | | PDK1 | |
| PER1 | | PGR | | PHF1 | | PHF6 | | PIK3C2A | | PIK3C2B | | PIK3C2G | |
| PIK3C3 | | PIK3CA | | PIK3CB | | PIK3CG | | PIK3R1 | | PIK3R2 | | PIM1 | |
| PKD1L2 | | PKHD1 | | PLAG1 | | PLCB1 | | PLCG1 | | PLCG2 | | PLK1 | |
| PLXNA1 | | PLXNB2 | | PMS1 | | PMS2 | | PNRC1 | | POLD1 | | POLE | |
| POLQ | | POM121 | | POM121L12 | | POT1 | | POU2AF1 | | PPM1D | | PPP1R17 | |
| PPP2R1A | | PPP6C | | PRDM1 | | PRDM16 | | PREX2 | | PRF1 | | PRKAA1 | |
| PRKAR1A | | PRKCB | | PRKCI | | PRKDC | | PRRX1 | | PRX | | PSG2 | |
| PSIP1 | | PSMB1 | | PSMB5 | | PTCH1 | | PTCH2 | | PTEN | | PTGS1 | |
| PTGS2 | | PTPN11 | | PTPN13 | | PTPN2 | | PTPRB | | PTPRD | | PTPRK | |
| PTPRO | | PTPRS | | PTPRT | | PTPRU | | RAB35 | | RAC1 | | RAC2 | |
| RAD21 | | RAD50 | | RAD51 | | RAD51B | | RAD51C | | RAD51D | | RAD52 | |
| RAD54B | | RAD54L | | RAF1 | | RANBP2 | | RARA | | RASA1 | | RASGRP1 | |
| RB1 | | RBL1 | | RBM10 | | RECQL | | RECQL4 | | REL | | RELN | |
| RET | | RFC1 | | RGS3 | | RHEB | | RHOA | | RHOH | | RHOT1 | |
| RICTOR | | RINT1 | | RIT1 | | RNASEL | | RNF43 | | ROBO1 | | ROBO2 | |
| ROBO3 | | ROCK1 | | ROS1 | | RPGR | | RPS6KB1 | | RPS6KB2 | | RPTOR | |
| RSPO2 | | RSPO3 | | RUNX1 | | RUNX1T1 | | RUNX2 | | RXRA | | RYR1 | |
| RYR2 | | SBDS | | SCUBE2 | | SDC4 | | SDHA | | SDHAF2 | | SDHB | |
| SDHC | | SDHD | | SEC31A | | SEMA3A | | SEMA3E | | SEMA6A | | SERPINA7 | |
| SERPINB3 | | SERPINB4 | | SETBP1 | | SETD2 | | SETDB1 | | SF1 | | SF3A1 | |
| SF3B1 | | SFPQ | | SGCZ | | SGK1 | | SH2B3 | | SH2D1A | | SH3PXD2A | |
| SHH | | SI | | SIN3A | | SLC16A1 | | SLC1A2 | | SLC22A16 | | SLC22A18 | |
| SLC22A2 | | SLC22A3 | | SLC34A2 | | SLCO1B3 | | SLIT1 | | SLIT2 | | SLX4 | |
| SMAD2 | | SMAD3 | | SMAD4 | | SMARCA4 | | SMARCB1 | | SMARCD1 | | SMARCE1 | |
| SMC1A | | SMC1B | | SMO | | SNCAIP | | SNTG1 | | SNX29 | | SOCS1 | |
| SOD2 | | SOS1 | | SOX10 | | SOX17 | | SOX2 | | SOX9 | | SPEN | |
| SPOP | | SPRR3 | | SPSB4 | | SPTA1 | | SRC | | SRD5A2 | | SRGAP1 | |
| SRGAP3 | | SRSF2 | | SRSF7 | | STAG1 | | STAG2 | | STAT1 | | STAT3 | |
| STK11 | | SUCLG1 | | SUCLG2 | | SUFU | | SULT1A1 | | SUZ12 | | SVEP1 | |
| SYK | | SYNCRIP | | SYNE1 | | TAF1 | | TAF15 | | TAF1L | | TAL1 | |
| TBL1XR1 | | TBX15 | | TBX22 | | TBX3 | | TCEB1 | | TCF12 | | TCF3 | |
| TCF4 | | TCF7L2 | | TCL1A | | TEC | | TENM3 | | TERC | | TERT | |
| TET1 | | TET2 | | TFDP1 | | TFDP2 | | TFE3 | | TGFBR1 | | TGFBR2 | |
| THBS2 | | TJP1 | | TLE1 | | TLL2 | | TLR4 | | TLX3 | | TMEM127 | |
| TMEM132D | | TMPRSS2 | | TNFAIP3 | | TNFRSF14 | | TNFSF11 | | TNN | | TOP1 | |
| TOP2A | | TP53 | | TP53BP1 | | TP63 | | TP73 | | TPM3 | | TPR | |
| TRAF2 | | TRAF7 | | TRIM24 | | TRIM58 | | TRIO | | TRPC5 | | TRRAP | |
| TSC1 | | TSC2 | | TSHR | | TSHZ2 | | TSHZ3 | | TTF1 | | TUBA3C | |
| TUBB3 | | TUSC3 | | TXNIP | | TYMS | | TYR | | U2AF1 | | UBE2D2 | |
| UBR5 | | UGT1A1 | | UMPS | | UPF3B | | USH2A | | USP6 | | USP8 | |
| VEGFA | | VEZF1 | | VHL | | VIM | | VTCN1 | | WASF3 | | WDR90 | |
| WDTC1 | | WHSC1 | | WHSC1L1 | | WIPF1 | | WNK1 | | WNT5A | | WRN | |
| WSCD2 | | WT1 | | WWOX | | WWP1 | | WWP2 | | XIAP | | XPC | |
| XPO1 | | XRCC1 | | XRCC2 | | XRCC3 | | YAP1 | | YY1AP1 | | ZBTB16 | |
| ZC3H11A | | ZFHX3 | | ZFP36L1 | | ZFP36L2 | | ZFPM2 | | ZIC3 | | ZMAT3 | |
| ZNF217 | | ZNF384 | | ZNF521 | | ZNF638 | | ZNF750 | | ZNF804B | |  | |
